# Hematologic markers of distant metastases and poor prognosis in gynecological cancers

**DOI:** 10.1186/s12885-019-5326-9

**Published:** 2019-02-12

**Authors:** O. Abu-Shawer, M. Abu-Shawer, N. Hirmas, A. Alhouri, A. Massad, B. Alsibai, H. Sultan, H. Hammo, M. Souleiman, Y. Shebli, M. Al-Hussaini

**Affiliations:** 1 University of Jordan School of Medicine, Amman, Jordan; 2 Prince Hamzah Hospital, Amman, Jordan; 3 King Hussein Cancer Center, Amman, Jordan; 4 Department of Pathology and Laboratory Medicine, King Hussein Cancer Center, Amman, 11941 Jordan

**Keywords:** Gynecological cancer. Neutrophil-lymphocyte ratio. Distant metastases. Survival

## Abstract

**Background:**

Despite the recent progress in the development of anti-cancer drugs, the treatment of metastatic tumors is usually ineffective. The systemic inflammatory response performs key roles in different stages of the carcinogenesis process including metastasis. The high neutrophil-lymphocyte ratio (NLR), monocyte-lymphocyte ratio (MLR), and platelet-lymphocyte ratio (PLR) were found to be associated with poor survival rates in the majority of solid tumors. However, only a few studies were conducted to further investigate this association in patients with advanced gynecological cancers.

**Methods:**

Clinical data from 264 patients with FIGO stage III and IV gynecological (endometrial, ovarian and cervical) cancers treated at King Hussein Cancer Center (Amman-Jordan) from 2006 to 2012 were retrospectively reviewed. We examined the association between absolute neutrophil count (ANC), absolute monocyte count (AMC), MLR, PLR, and NLR with distant metastases, overall survival and event-free survival in gynecological cancers. For survival analysis, Receiver Operating Characteristic (ROC) curve analysis was operated to determine the optimal cutoff values.

**Results:**

Patients with high baseline NLR (≥4.1) had more baseline distant metastases than patients with low baseline NLR (< 4.1), (*p*-value 0.045). Patients with high baseline AMC (≥560) had more distant metastases in comparison to patients with low baseline AMC (< 560), (*p*-value 0.040). Furthermore, Patients with high baseline PLR (≥0.3) had more distant metastases in comparison to patients with low baseline PLR (< 0.3), (*p*-value 0.025). Additionally, patients with high baseline ANC (≥5700) had worse overall survival compared to the patients with low baseline ANC (< 5700), (*p*-value 0.015). Also, patients with high baseline AMC (≥490) had worse overall survival compared to the patients with low baseline AMC (< 490), (*p*-value 0.044).

**Conclusion:**

Different hematologic markers obtained from a cheap test (CBC) could potentially be used to predict the presence of distant metastases thus used as prognostic indices in gynecological cancers.

## Background

The malignant tumors of the female genital system; cervical cancer (cancer of cervix uteri), endometrial cancer (cancer of corpus uteri), ovarian cancer, fallopian tube cancer, vaginal cancer, vulvar cancer, and choriocarcinoma are a major cause of morbidity and mortality all over the world. [1]

The immune system and the inflammatory response play a key role in different stages of the carcinogenesis process including initiation, invasion, promotion and metastasis. [2] Many studies have shown that several inflammatory markers including absolute neutrophil count (ANC), absolute monocyte count (AMC), neutrophil-lymphocyte ratio (NLR), monocyte-lymphocyte ratio (MLR), and platelet-lymphocyte ratio (PLR) are associated significantly with worse prognosis of different types of solid tumors including cervical, endometrial and ovarian cancers. [3–5] A high NLR is detected when the absolute neutrophil count is high and the absolute lymphocyte count is low. Though it remains poorly understood why a high NLR is associated with dismal prognosis in different types of solid tumors [4–13], it may be explained by the neutrophils’ role in metastasis promotion via releasing certain growth factors such as VEGF and specific proteases. [14] Also, lymphopenia (low Lymphocyte count) may influence their essential role in fighting tumor cells by inducing cytotoxic cell death and impeding tumor cell proliferation and migration, thus diminishing the immune response to malignancy. [15] Interestingly, recent studies suggested that a high NLR is also an independent predictive factor for the baseline presence and subsequent development of brain metastasis in advanced non-small cell lung cancer (NSCLC) and for the baseline presence and subsequent development of lung metastasis in stage IV colorectal cancer (CRC). [16, 17] The value of NLR as a predictive marker for the identification of metastasis may apply also to other solid tumors like cervical, endometrial and ovarian cancers.

Metastasis is considered the leading cause of the majority of cancer-related deaths among patients with various types of malignancy. [18] A significant proportion of patients diagnosed with gynecological cancers have distant metastases at the time of diagnosis, and this proportion has been declining over the last several years. [19] However, the overall survival (OS) of all patients with metastatic gynecological malignancies regardless of the primary origin is grim and the main aim of treatment is to lengthen survival while maintaining an appropriate quality of life. [20] Early detection of metastasis is important to identify patients with limited disease who potentially could benefit from a more aggressive approach aiming to stop micro-metastatic lesions from progressing into an uncontrollable macro-metastases. Therefore, looking for new biomarkers for detecting the commencement of the metastatic process can decrease the mortality among gynecological cancer patients by encouraging a more intensive imaging surveillance and considering other preventive strategies.

Although the biological behavior and the prognostic value of NLR were tremendously researched, no study has been conducted to explore the association between NLR and other hematologic markers with the distant metastases of cervical, endometrial and ovarian cancers. In this retrospective study, we aim to explore the relationship between the peripheral counts of immune cells and distant metastases in gynecological cancers as this will have important implications in the management of patients with advanced gynecological cancers. Do FIGO stage IV endometrial, ovarian and cervical cancer patients have higher ANC, ALC, AMC, NLR, MLR, and PLR valuesthan FIGO stage III patients at the time of presentation?

## Methods

This is a retrospective chart review study that was approved by the Institutional Review Board office at King Hussein Cancer Center (KHCC). We included 264 patients diagnosed with stage III or IV gynecological cancer, as confirmed by histopathology and/or radiology reports, who received their treatment for cancer at KHCC (Amman, Jordan) between 2006 and 2012. Chest, abdomen, and pelvic computed tomography (CT) scans have been used to detect distant metastases (lung, liver, rectum, and bladder). Clinical data including the age, histology, and location of the tumor were assessed for possible correlation with the baseline presence of distant metastases.

The results of the routinely obtained complete blood count (CBC), at the time of diagnosis before initiation of any treatment were reviewed. Total white blood cells (WBC) counts and the individual counts of each component including neutrophils, lymphocytes, monocytes, and platelets were also obtained. The pre-treatment baseline NLR, MLR and PLR have been calculated using these formulas; NLR = ANC/ALC, MLR = AMC/ALC and PLR = Platelet Count/ALC, respectively.

The Receiver Operating Characteristic (ROC) curve was used to determine the optimal cut-off value of ANC, AMC, ALC, MLR, and PLR for the survival analysis matching the most extreme joint sensitivity and specificity. *P*-value of ≤0.05 was determined as the cut off for significant association.

The statistical analysis consists of two phases. In the first phase, we examined the predictive value of the peripheral count of each type of immune cells at presentation for the presence of distant metastases in gynecological cancers by examining the association between the baseline ANC, AMC, ALC, NLR, MLR, and PLR with the baseline presence of distant metastases. We also examined the association between baseline presence of distant metastases with the other relevant clinical variables such as the age, location of the primary tumor and the histological subtype.

In the second phase, after determining the optimal cutoff value for association with OS using a ROC curve analysis corresponding to maximum joint sensitivity and specificity for all hematologic markers, we assessed the prognostic value of the peripheral counts of each type of immune cells at presentation in gynecological cancers by examining the association between the baseline ANC, AMC, ALC, NLR, MLR, and PLR with the OS and with the event free survival (EFS). We did not examine the prognostic impact of other clinical factors such as the age, the histologic subtype and the pathologic grade in gynecological cancers because it is well-documented in the medical literature.

We did not analyze the data separately based on the tumor type because we focused on identifying the relationship between the peripheral counts of immune cells and the presence of distant metastases. The systemic inflammatory response to the metastatic lesion does not vary between solid tumors.

## Results

The clinical features of 264 FIGO stage III and IV patients with gynecological cancers are summarized in Table 1. The age ranged from 16 to 94 years, median: 56 years, and the mean: 57 years. Seventy-two (27%) patients had cervical cancer, 76 (29%) patients had endometrial cancer, and 116 (44%) patients had ovarian cancer. One-hundred and thirty (49%) patients were in FIGO stage III while 134 (51%) patients were in FIGO stage IV at time of diagnosis. The mean of baseline NLR was 4.1 (median: 3.3 & range: 0.24–18), while the mean of MLR was 0.4 (median: 0.29 & range: 0.014–2). The mean of PLR was 0.3 (median: 0.2 & range: 0.04–1.9). The mean of ANC was 6080 and the median 5500. The mean of ALC was 1800 and the median 1700. The mean of AMC was 560 and the median 530.

**Table 1:** the clinical features of the cohort of patients.

Patients features	No. of patients (%)
Age, median (range)	56 (16–94 years)
Histologic subtype:	
Adenocarcinoma	197 (75%)
Squamous carcinoma	67 (25%)
FIGO Stage:	
3A	43 (16%)
3B	35 (13%)
3C	52 (20%)
4A	90 (34%)
4B	44 (17)
Location	
Cervix	72 (27%)
Endometrium	76 (29%)
Ovary	116 (44)
Hematologic Parameters:	Mean (Median)
ANC	6080 (5500)
ALC	1800 (1700)
AMC	560 (530)
NLR	4.1 (3.3)
MLR	0.4 (0.29)
PLR	0.3 (0.2)

Patients with high baseline NLR (≥4.1) had more distant metastases (FIGO Stage IV) in comparison to patients with low baseline NLR (< 4.1), (*p*-value 0.045, OR: 1.7, CI: 1.0–2.8) (Table 2). Patients with high baseline PLR (≥0.3) had more distant metastases (FIGO Stage IV) in comparison to the patients with low baseline PLR (< 0.3), (*p*-value 0.025, OR: 1.2, CI: 0.7–2.1) (Table 2). Furthermore, patients with high baseline AMC (≥560) had more distant metastases (FIGO Stage IV) in comparison to the patients with low baseline AMC (< 560), (*p*-value 0.040, OR: 1.5, CI: 0.9–2.4) (Table 2). The results showed that other baseline parameters ANC, ALC, and MLR were not associated with the presence of distant metastases (FIGO Stage IV) (*p*-value: 0.18, 0.093, and 0.78 respectively).

**Table 2:** The association between the distant metastases with baseline NLR

	Distant metastases				
	Present	Absent	*p*-value	OR	CI
NLR ≥ 4.1	51 (58.6%)	36 (41.4%)	0.045	1.7	(1.01–2.8)
NLR < 4.1	80 (46.0%)	94 (54.0%)			
PLR ≥ 0.3	35 (53.8%)	30 (46.2%)	0.025	1.21	(0.7–2.1)
PLR < 0.3	96 (49.0%)	100 (51.0%)			
AMC ≥ 560	84 (54.2%)	71 (45.8%)	0.04	1.5	(0.9–2.4)
AMC < 560	47 (44.3%)	59 (55.7%)			

In univariate analysis, tumor location (cervix, endometrium and ovary), age at time of diagnosis, as well as the histologic subtype (adenocarcinoma and squamous) were not associated with baseline presence of distant metastases (FIGO stage IV), (*p*-value 0.4, 0.17, and, 0.41, respectively).

**Fig. 1:**
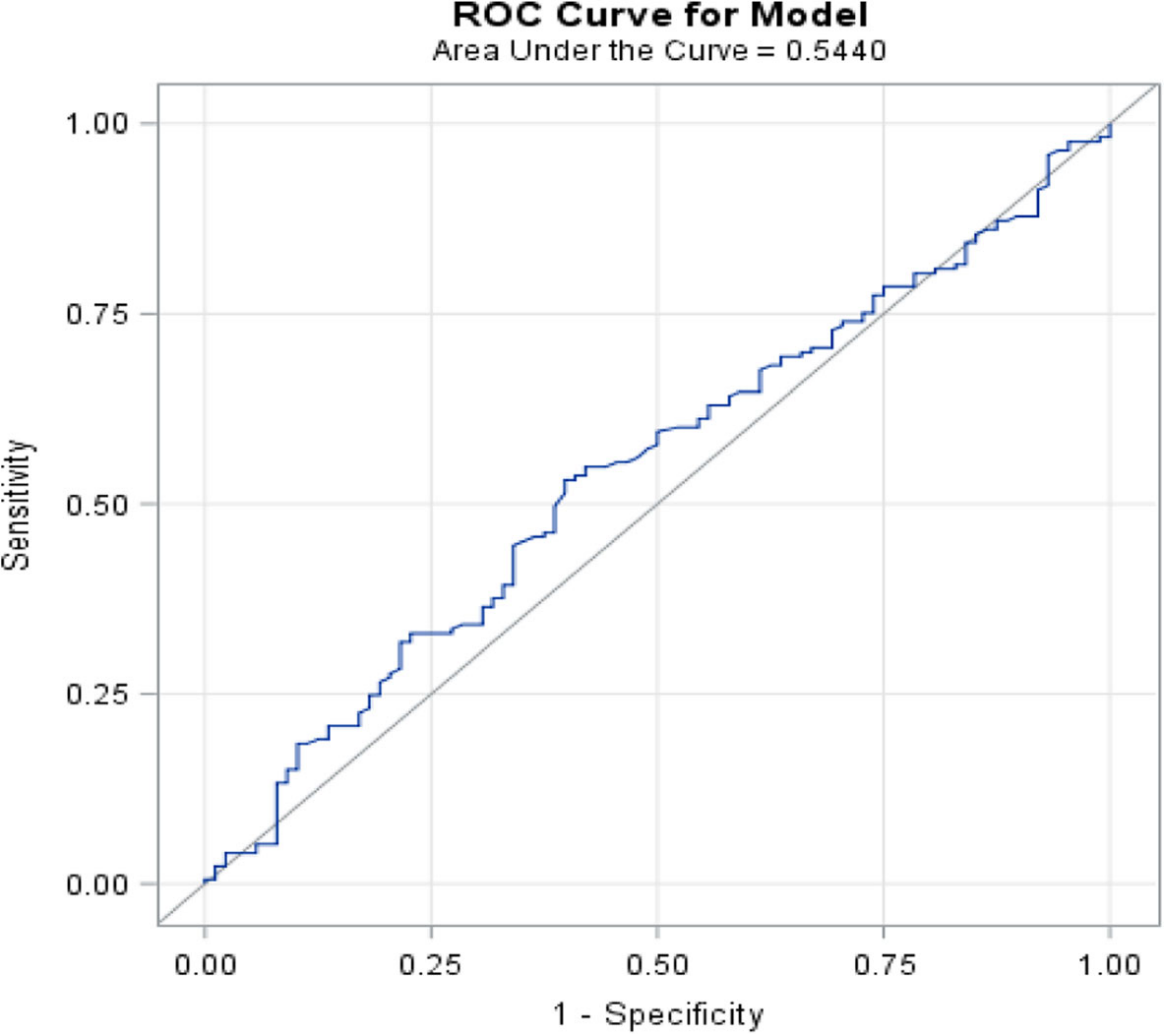
Receiver-operating-characteristic (ROC) and area under the curve (AUC) for the ANC

**Fig. 2:**
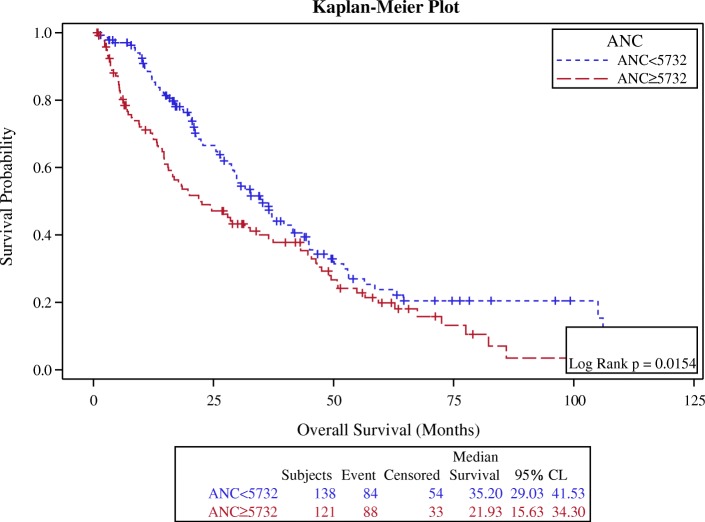
Kaplan Meier curve for overall survival for patients with ANC ≥ 5700

**Fig. 3:**
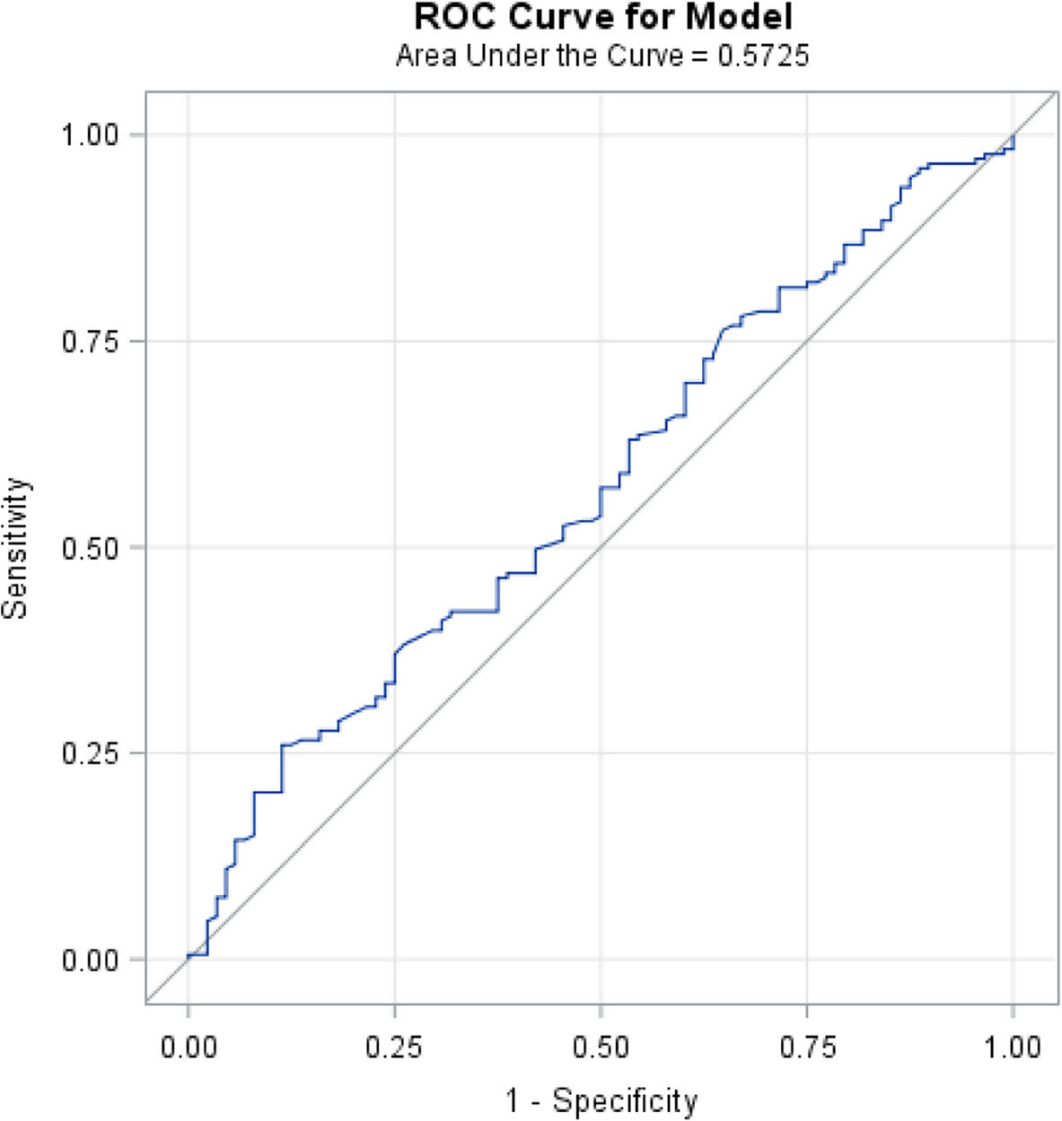
Receiver-operating-characteristic (ROC) and area under the curve (AUC) for the AMC

**Fig. 4:**
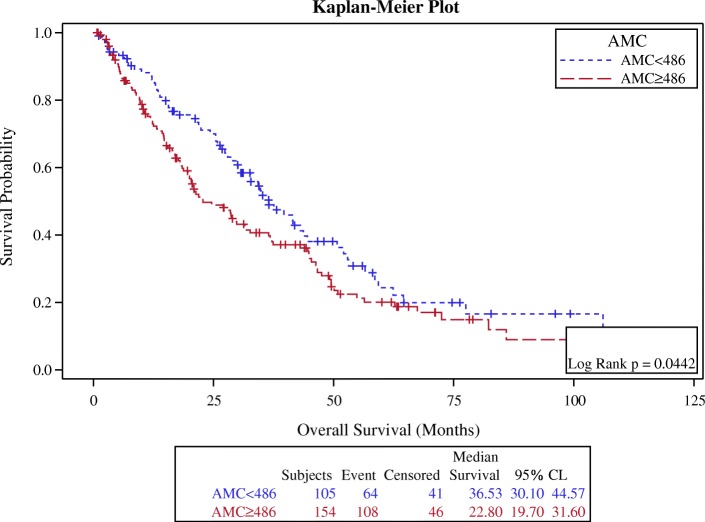
Kaplan Meier curve for overall survival for patients with AMC ≥ 490

In survival analysis, the optimal ANC cutoff value was determined as 5700, an area under the curve AUC recorded as 0.5440 (Fig. 1). We found that patients with high baseline ANC (≥5700) had worse overall survival in comparison with the patients with low baseline ANC (< 5700), (*p*-value 0.015, HR: 1.5, CI: 1.1–1.9) (Fig. 2, Table 3). Also, the optimal AMC cutoff value was determined as 490, an area under the curve AUC recorded as 0.5725 (Fig. 3). Patients with high baseline AMC (≥490) had worse overall survival in comparison with the patients with low baseline AMC (< 490), (*p*-value 0.044, HR: 1.4, CI: 1.01–1.8) (Fig. 4, Table 3). The optimal cutoff values for other hematologic parameters ALC, MLR, and PLR were determined as 1700, 0.3, and 0.2, respectively. ALC, MLR, and PLR were not associated with the overall survival, (*p*-value 0.054, 0.055, and 0.6, respectively) (Table 4).

**Table 3:** The association between OS with baseline ANC & AMC

	Median OS	Number of cases	*p*-value	HR	CI
ANC ≥ 5700	22 months	138 (54%)	0.015	1.5	(1.1–1.9)
ANC < 5700	35 months	120 (46%)			
AMC ≥ 490	23 months	154 (60%)	0.044	1.4	(1.01–1.8)
AMC < 490	37 months	105 (40%)

**Table 4:** The association between OS with the different hematological variables

	*p*-value	HR	CI
MLR ≥ 0.3	0.055	1.3	(0.9–1.8)
MLR < 0.3			
PLR ≥ 0.2	0.6	1.1	(0.8–1.5)
PLR < 0.2			
ALC ≥ 1700	0.054	1.3	(0.9–1.8)
ALC < 1700			
ANC ≥ 5700	0.015	1.5	(1.1–1.9)
ANC < 5700			
AMC ≥ 490	0.044	1.4	(1.01–1.8)
AMC < 490			

In addition, we found that these hematologic indices ANC, AMC, ALC, MLR, and PLR were not associated with event free survival (*p*-value 0.15, 0.6, 0.77, 0.17, 0.23, respectively) (Table 5).

**Table 5:** The association between EFS with the different hematological variables

	*p*-value	HR	CI
MLR ≥ 0.3	0.17	1.2	(0.9–1.6)
MLR < 0.3			
PLR ≥ 0.2	0.23	1.1	(0.9–1.4)
PLR < 0.2			
ALC ≥ 1700	0.77	1.04	(0.8–1.4)
ALC < 1700			
ANC ≥ 5700	0.15	1.2	(0.9–1.6)
ANC < 5700			
AMC ≥ 490	0.6	1.07	(0.8–1.4)
AMC < 490			

## Discussion

In this study, we assessed the potential value of some hematological indices for predicting the baseline presence of distant metastases in patients with gynecological cancers (endometrial, ovarian and cervical). An elevated baseline NLR (≥ 4.1) was an independent factor of the baseline presence of distant metastases. In addition, the results showed that other hematologic indices such as AMC and PLR were associated significantly with distant metastases (FIGO stage IV) in gynecological cancers.

To the best of our knowledge, this appears to be the first description of the potential predictive value of these hematologic parameters for detecting distant metastases. These markers are easily obtained from an affordable test; the CBC, which is done routinely during the management and follow-up of all patients with malignancy. Using simple tests such as CBC to predict the presence of distant metastases might have direct impact on gynecological cancers patient care. Surveillance for distant metastases through more frequent imaging, and consideration for prophylactic strategies in patients with high NLR could be an attractive area for future research.

The relationship between systemic inflammatory response and cancer is well documented in the literature. [21, 22] Cancer cells play a vital role in stimulating the release of granulocyte colony-stimulating factor (GCSF) that can increase the peripheral count of neutrophils. On the other side, neutrophils play a vital role in cancer cells invasion and metastasis by releasing certain growth factors such as VEGF and other proteases such as matrix-metalloproteinase and elastase enzymes. [11, 23] Monocytes can also stimulate cancer cells migration and inhibit antitumor immunity. [24–26] In contrast, lymphocytes have a key role in supplying antitumor immunity explaining the association between low lymphocyte count with poor outcome in cancer. [21] High lymphocyte count was shown to be  a good prognostic marker in patients with different types of solid tumors. [27–29]

Several retrospective studies delineated the significance of NLR changes as prognostic tools. [30, 31] One study reported that early drop of NLR correlated with an excellent outcome in patients with metastatic stage IV renal cell carcinoma. [30] Another study reported that post-treatment NLR changes can be used to predict the recurrence in patients with renal tumors. [31]

Using glucocorticoids prior to the date of measuring baseline NLR may be a confounding factor. Actually, extreme and persistent leukocytosis can be induced by administration of small doses of glucocorticoids. [32] Glucocorticoids can cause neutrophilia by pushing neutrophil cells from the marginated pool into the main bloodstream pool. [33] Therefore, we excluded all patients taking glucocorticoids to avoid this confounding effect. However, there are other factors that may affect and alter the CBC results, like systemic inflammatory conditions and infection, which could not have been controlled in this retrospective study.

Limitations of this study include the retrospective nature of the study with all data collected at a single institution. There was no stratification by the number of metastatic lesions. No information about confounding factors that might alter the peripheral counts of immune cells such as infections at the time of diagnosis. Also, large lung, liver, and bladder metastases (greater than 1–2 cm in size) could be detected by the optimum quality CT techniques with a high level of accuracy. In contrast, microscopic metastases (smaller than 1–2 mm in size) are rarely detected by anatomic imaging methods therefore, patients who developed metastasis after treatment, might have had small, tiny, and non-measurable metastases not being detected by the initial CT scan.

## Conclusion

Markers includig NLR, AMC, and PLR may assist identifying patients more prone to develop distant metastases. Surveillance for distant metastases by frequent imaging and prophylactic treatment measures in this special group could potentially improve the final outcome and quality of life. High baseline ANC and AMC appear to be associated with poor OS. Prospective studies are needed to confirm the value of these blood markers in predicting the presence of distant metastases and the in patients with gynecological cancers.

## Abbreviations

ALC: Absolute Lymphocyte Count
AMC: Absolute Monocyte Count
ANC: Absolute Neutrophil Count
CBC: Complete Blood Count
EFS: Event-Free Survival
FIGO: International Federation of Gynecology and Obstetrics
MLR: Monocyte-Lymphocyte Ratio.
NLR: Neutrophil-Lymphocyte Ratio.
OS: Overall Survival.
PLR: Platelet-Lymphocyte Ratio.
